# Medical students as sexual health peer educators: who benefits more?

**DOI:** 10.1186/1472-6920-14-162

**Published:** 2014-08-07

**Authors:** Florence Bretelle, Raha Shojai, Julie Brunet, Sophie Tardieu, Marie Christine Manca, Joelle Durant, Claire Ricciardi, Leon Boubli, George Leonetti

**Affiliations:** 1 Department of Gynaecology and Obstetrics, Gynepole Marseille, AP-HM, AMU, Division of Women and Child Care, Hôpital Nord, Chemin des Bourelly 13915, Marseille Cedex 20, France; 2 UMR CNRS-IRD 6236 –Marseille Faculty of Medicine, Aix Marseille Université, Marseille INSERM U1095, France; 3 CIC 1409, Assistance Publique des Hôpitaux de Marseille (AP-HM), Aix Marseille Université, AMU, Hôpital de la Conception, 147 bd Baille, 13005 Marseille, France; 4 Medical Evaluation, Department of Public Health, Assistance Publique-Hôpitaux de Marseille, Aix Marseille Université, Marseille, France; 5 Department of Medical Genetics, l’Hôpital d'Enfants de la Timone, Marseille, France; 6 National Education Authority of Aix-Marseille, Education Nationale, Aix-en-Provence, France; 7 Family Planning of Bouches-du-Rhône, Marseille, France; 8 Faculty of Medicine, Aix Marseille Université, AMU, Marseille, France

**Keywords:** Education, Reproductive health, Public health, Teenagers

## Abstract

**Background:**

A prospective study was conducted to evaluate the impact of an educational reproductive health program on medical student peer educators and the secondary school pupils whom they taught.

**Methods:**

The Marseille School of Medicine and ten public secondary schools participated in the study. Medical students were recruited and trained as peer educators to promote sexual health in the secondary schools. The medical students and secondary school pupils were evaluated before and after education program. The main outcome measure was the sexual health knowledge score on a 20-item questionnaire (maximum score 20).

**Results:**

A total of 3350 students attended the peer-led course conducted by 107 medical students. The medical students’ score increased significantly before and after the course (from 15.2 ± 1.8 to 18.3 ± 0.9; p < 0.001). The knowledge score of the pupils increased (from 7.8 ± 4 to 13.5 ± 4.4; p < 0.001). The girls’ score was significantly higher than the boys’ score after the course, but not before (14.5 ± 3.3 vs 12.5 ± 4.6; p < 0.001). Prior to the course, the score among the female medical students was significantly higher than that of the males. The overall knowledge increase was not significantly different between medical students and secondary school pupils (mean 3.1 ± 1 and 5.7 ± 4 respectively; p > 0.05).

**Conclusions:**

The program was effective in increasing the knowledge of medical students as well as secondary school pupils. Male sexual health knowledge should be reinforced.

## Background

The rate of abortions among teenagers in France has dramatically increased in recent years, increasing from 8.9 to 14.5 per 1000 women between 2002 and 2012 [1]. Currently, it is estimated that over 26 000 adolescents have an abortion annually, representing 15% of pregnancy terminations [1]. Increased efforts are being made to improve reproductive health programs, though their implementation remains controversial [2,3]. The increase of unintended pregnancies may reflect the limited effectiveness preventive measures delivered in secondary schools.

In France, the content and quality of these educational programs are varied and their impact in curbing the rate of unintended pregnancies is difficult to assess since they are provided by a wide range of professionals, including biology teachers, school nurses, social health workers and medical doctors specialised in public health, family planning, general medicine or gynaecology. The initial training of these professionals varies widely, and the content of the message given to pupils is difficult to summarize, and therefore evaluate. The methods of communication are diverse and have not been well evaluated.

A different approach involving peers as sex educators has recently been implemented in the UK. Recent studies of peer education programs have shown positive impact on risk-taking behaviours compared to traditional teacher-led educational programs. Peer education is a broad concept with several definitions. In brief, peer education programs train and motivate young people to undertake informal or organized educational activities with their peers with the aim of increasing their knowledge in order to decrease risky behaviour and improve future health. This concept has been further extended to training medical school students as peer educators in settings where sexual health education did not exist, and the results have been encouraging [4-6]. The peer educators have the potential advantage to have less stereotype beliefs and were all given the same training at the beginning of the program. They were also younger, and as such were able to communicate more easily with the pupils [7]. On the basis of these models, medical students were recruited as peer educators to promote sexual health in French secondary schools. Medical students have the advantage of their medical knowledge and are slightly older than the usual peer educators. Students themselves can also be a target of sexual health program, as it has been recently reported high rate of risky sexual behaviours [8].

Therefore our aim was to determine whether an educational reproductive health program conducted by medical student peer educators increased the knowledge of secondary school pupils and also of the medical students themselves.

## Methods

A prospective evaluation of a new program on sexual health was conducted between September 2010 and June 2012 (two academic years).

The program intents to develop a strong collaboration between the professional routinely involved in the information on sexual health in the country to educate the medical student in this specific field.

The School of Medicine of the University of Aix-Marseille II created a specific course entitled “Sexual Education Health Care and Contraception” in 2006 for second- and third-year medical students. The program had been formulated over the course of a year by a multidisciplinary team of professionals brought together by academic faculty members (FB, RS), and included representatives of family planning associations (MCM and CR) and from the local education authority (JD). The French ministry of education approved the educational content.

Registration for the course was optional, but provided bonus points were offered at the end of the year to those completing the medical curriculum. The local ethics committee of Institut Federatif de Recherche (IFR) 48 approved, validated and registered the project under the number 10–008.

The program provided 24 hours of instruction on reproductive preventive care, counselling, communication and public health; and was delivered over the course of six afternoons. Following the didactic course, medical students were divided in groups of two (a female and a male, or two females). Each pair conducted 6 educational sessions for the 8^th^ grade classes in various secondary schools. Fourteen secondary schools in the town of Marseille were contacted in order to participate in the program, and 10 voluntarily agreed to do so. Each session was divided into two parts: the first with the class as a whole, and then they were divided into same-sex groups for peer and pupil discussion.

The medical students were paired (before and after questionnaire) for analysis. For the interventions in the school, the students were grouped (by 2 or 3) and if possible with a female and a male.

Medical students taught on matters concerning sexuality, unwanted pregnancy, contraception and infectious diseases. Pupils could also submit questions written anonymously. The students had also the possibility to draw on the dashboard.

The teenagers’ parents had previously been informed by the school about the peer-led sex education program, and they were given the option of refusing the program; in the which case, the pupil went to study in another room.

Evaluation was performed by an independent team from the Public Health Department of the university hospital (ST) and covered the 2007–2008 and 2008–2009 academic years. Medical students and secondary school pupils took one questionnaire that evaluated their knowledge of sexual health before and after the course, and another that evaluated their degree of satisfaction with the peer-led sessions.

Medical students’ answers to the questionnaires did not influence their grade upon completion of the course.

The knowledge questionnaire included 20 items focused on contraception, abortion, sexuality, and infections (Table 1). Medical students’ and pupils’ performance was graded on a scale from 0 to 20 (one point per correct response). Higher scores indicated high levels of knowledge. The knowledge questionnaire items were the same for the secondary school pupils and medical students. The satisfaction questionnaire was a 10-item, 5-point Likert scale self-report measure. This questionnaire focused on program organization, content and overall satisfaction. The satisfaction questionnaire items were different for pupils and medical students.

**Table 1:** Questionnaire fullfilled by students and pupils before and after the intervention

**Age :**	**Sex :**	**Classroom :**	
	**True**	**False**	**I don’t know**
1. The risk of pregnancy is low when the male withdraws before ejaculation	❍	❍	❍
2. It is possible to become pregnant when having intercourse during menses	❍	❍	❍
3. If you avoid having sex during the ovulation period, the pregnancy risk is low	❍	❍	❍
4. It is possible to obtain contraceptive pills without parental consent	❍	❍	❍
5. Contraceptive pills are is available freely in the family planning clinic	❍	❍	❍
6. It is mandatory to have a gynaecologic examination before starting contraceptive pills	❍	❍	❍
7. If you forget your pill one more than 12 hours day you will not be protected from becoming pregnant	❍	❍	❍
8. When you stop taking contraceptive pills, the protective effect remains for several weeks	❍	❍	❍
9. Contraceptive pills can lead to infertility	❍	❍	❍
10. Condoms are effective in preventing sexually transmitted infections	❍	❍	❍
11. It is rare for condoms to break	❍	❍	❍
12. The best contraceptive method is a combination of condoms and contraceptive pills	❍	❍	❍
13. Minors can obtain the emergency pill anonymously and at no cost at family planning clinics and in schools	❍	❍	❍
14. The emergency pill is freely available in the pharmacy	❍	❍	❍
15. The emergency pill is more effective if it is administered soon after the unsafe intercourse	❍	❍	❍
16. Repeated use of the emergency pill is less effective than the classic contraceptive pill	❍	❍	❍
17. Intrauterine contraceptive devices are only for women who have already given birth	❍	❍	❍
18. Other available methods of contraception are the patch, ring, and contraceptive implant	❍	❍	❍
19. Contraception is a matter that also concerns males	❍	❍	❍
20. Pregnancy can occur even after the first act of intercourse	❍	❍	❍

**Figure 1:**
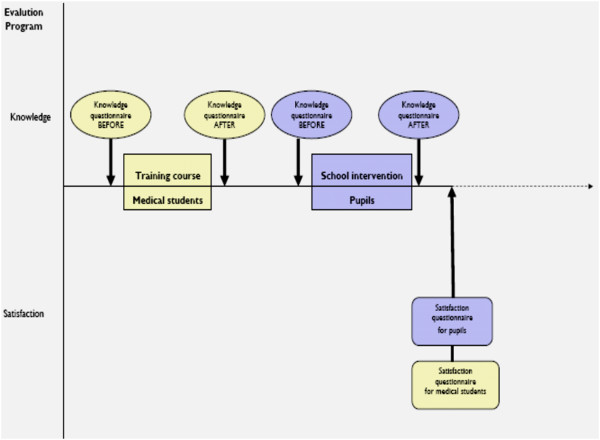
Evaluation chart.

Medical students completed 4 questionnaires: two knowledge questionnaires--one before and one after the didactic course and school sessions respectively, and the two other questionnaires evaluating their satisfaction with the program itself and their interventions.The pupils completed two questionnaires on the day of intervention in their school (before and after intervention). The evaluation chart is summarized in Figure 1. Open answer was available at the end of the questionnaire. They can describe freely in it their difficulties during their intervention.

All of the medical students and pupils were asked to fill out the questionnaires. Descriptive statistics were summarized by frequency or by mean ± standard deviation for quantitative parameters.

The scores on the knowledge questionnaire before and after the course were compared using a paired Student’s t-test for pupils and by Wilcoxon signed-rank test for medical students. The scores on the knowledge questionnaire were compared between gender using the Mann–Whitney test (medical students) or Student’s t- test (pupils) according to the distribution of the data. Satisfaction questionnaires were descriptively analysed. Lastly, the difference in overall knowledge acquisition between students and pupils was compared. Statistical analysis was performed using SPSS software. General level of significance was fixed at 0.05.

Power calculation: we estimate that pupils will have an initial correct response rate of 50% and that after intervention this rate will increase up to 60%. Thus with a study power of 80%, a sample size of 383 pupils was required. Therefore a quarter of questionnaires were randomly selected.

This program received a grant from the Bouches-du-Rhone General Counsel (Conseil General de Bouches du Rhone).

## Results

A total of 3350 pupils participated in the peer-led course. Sixty-five per cent of the questionnaires were correctly completed (before and after the program) and analysed (n = 2177 questionnaires were correctly fulfilled). Among them 25% were randomly selected (n = 544).

**Figure 2:**
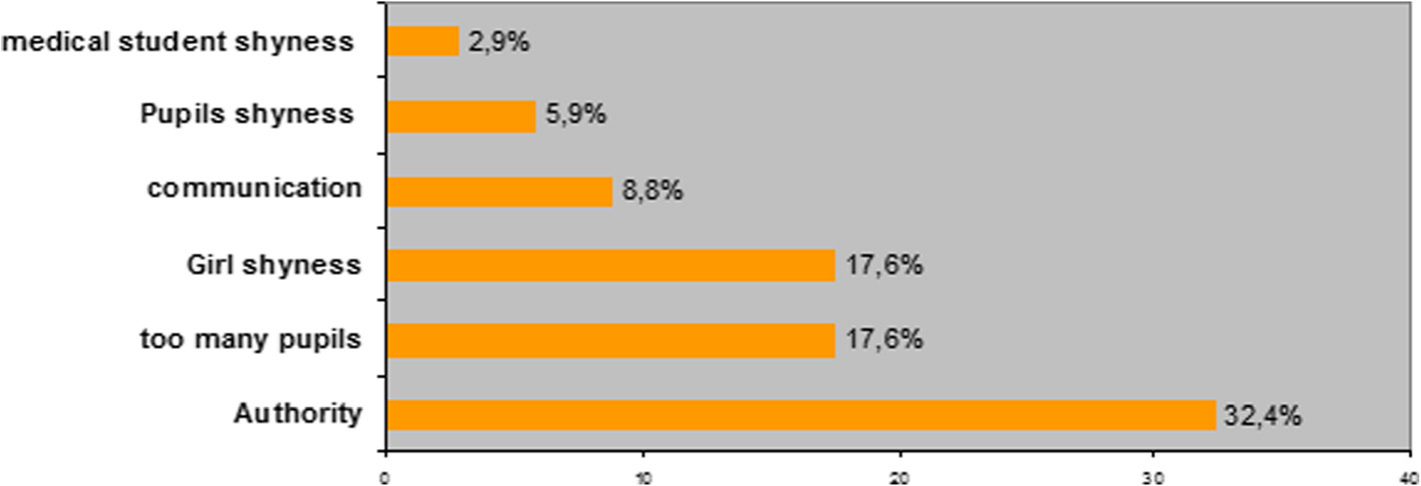
Difficulties during the course.

One hundred and seven medical students took the training course, and all filled out the evaluation forms. They were 21.2 (±2.8) years old, 51% were male and 58% were in their 3^rd^ year of medical school. The percentage reporting that they had received enough information for their own sexual health education was 96.8%. A total of 87.1% thought the program was important for their medical curriculum, and 90% pointed out the importance of practical application after the didactic course.The difficulties faced by the medical students are summarized in Figure 2. The main topics of their classes were contraception (94.1%), emergency contraception (91.2%), sexuality (91.2%), sexually transmitted infections including AIDS (82.4%), homosexuality (70.6%), abortion (70.6%), pornography (58.8%), human papilloma virus vaccination (58.8%), assisted reproductive techniques (26.5%), cervical cancer screening (17.6%) and parenthood (14.7%). Less frequent topics were adolescence, puberty, pregnancy, sexual violence, rape, male–female relationships, and sexual pleasure. Overall, 74.2% of the medical students reported being very satisfied with the course and 24.8% reported being satisfied. The percentage willing to repeat the experience was 83.9%.

**Figure 3:**
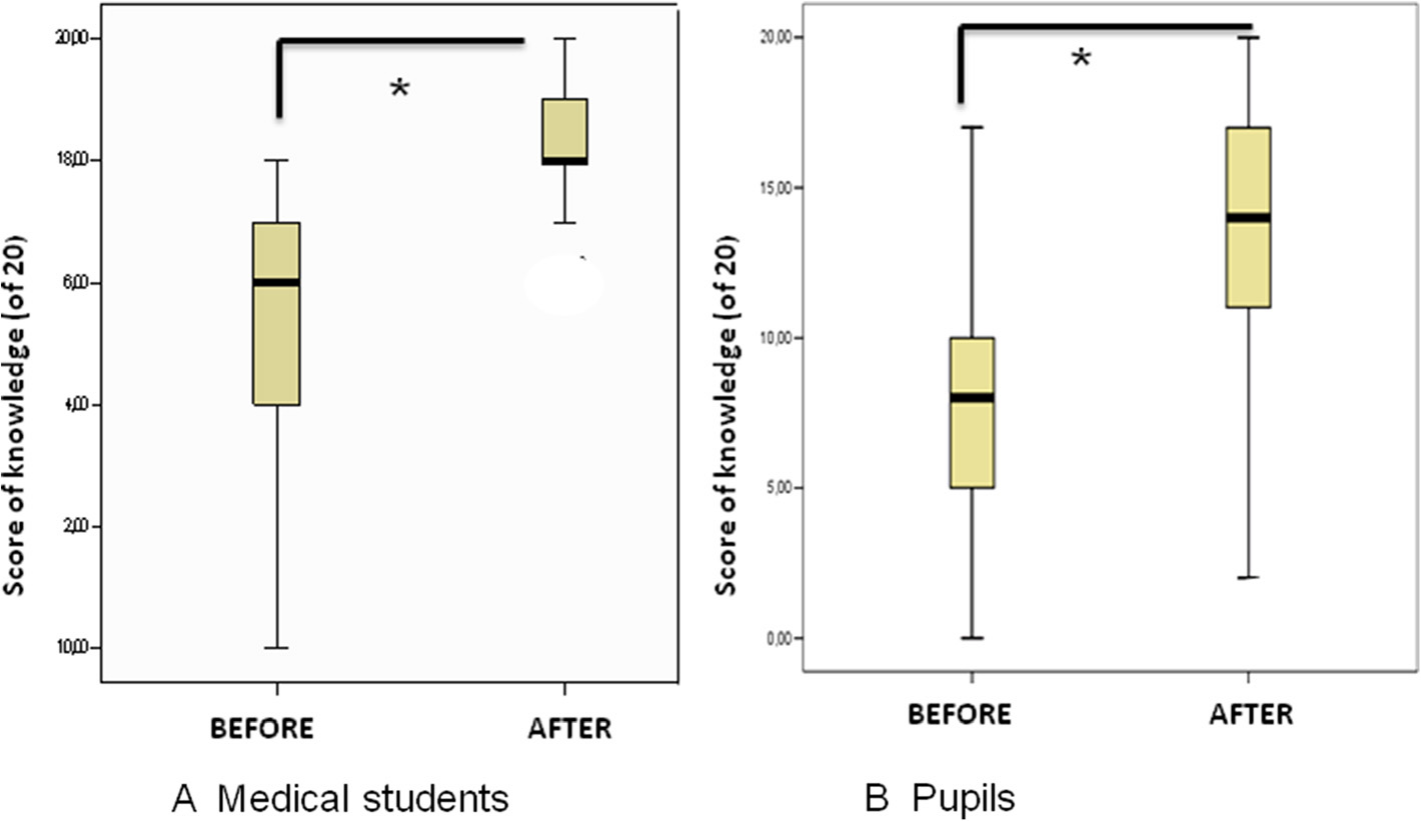
**Knowledge evaluation: A**: For Medical students before and after training course (maximum score of 20). P<0.05; **B**: For Pupils before and after course (maximum score of 20) P<0.05.

The secondary school pupils were 13.5 (±0.7) years old and 53.9% were male. A total of 87% were very pleased with the course. While 94.3% acknowledged having learned about sexual health education issues, 22.3% reported some form of embarrassment concerning various topics. A total of 78.4% requested similar peer-led courses on other topics such as: nutrition, addictions and driver safety. Although 81.6% of pupils discussed the course with their friends, only 39.4% discussed it with their parents.There was an increase in sexual health knowledge for both the medical student group and secondary school pupil group before and after the course (Figure 3). Before the didactic course, the medical students’ score in reproductive health was 15.2 (±2.0; out of 20). The females’ score was significantly higher compared to the males’ score (15.8 ± 1.8 vs14.5 ± 2.1; p < 0.001). The most common misconceptions were that intrauterine devices are reserved only for multiparous women (36% responded correctly) and there was no risk of pregnancy during menstrual periods (36% responded correctly).

After the course, the medical students’ score increased significantly (to 18.3 ± 0.9; p < 0.001), with no difference between the males and females (18.5 ± 0.2 vs 18.1 ± 0.4; p > 0.05).

Before the course, the, pupils’ score in reproductive health was 7.8 ± 4 (out of 20) with no difference between girls and boys (7.6 ± 4.1 vs 8.4 ± 3.8; p > 0.05). The most common misconceptions were that: a gynaecologic pelvic examination is mandatory before prescription of oral contraceptives (20% responded correctly), IUDs are reserved for multiparous women (20% responded correctly), the risk of pregnancy is low if coitus is interrupted before ejaculation (35% responded correctly), the risk of pregnancy is low if intercourse occurs outside the ovulation time frame (48% responded correctly), and emergency pills is freely and readily available (50% responded correctly).

After the course, the pupils’ knowledge score increased (to 13.5 ± 4.4; p < 0.001). The girls’ score was significantly higher than the boys’ score (14.5 ± 3.3 vs 12.5 ± 4.6; p < 0.001).

The facts that most pupils learned were that prolonged intake of oral contraceptives does not lead to infertility, a pelvic examination is not mandatory before intake of oral contraceptives, and there is a risk of pregnancy even if there is no ejaculation. However, following the course, 35% of pupils still thought that there was no time restriction for taking emergency hormonal contraception and 33% believed that contraception was a matter that concerned only the girls.

The overall increase in the knowledge score was not significantly different between medical students and pupils (score increases: + 5.7 ± 4 and 3.1 ± 1 respectively; p > 0.05).

## Discussion

Medical students as peer educators are effective in helping to increase teenagers’ knowledge in reproductive health issues in the short term. As with other classical peer-led programs, education by medical students is popular among pupils. An unexpected finding in our study was that the baseline level of reproductive health knowledge among medical students was low (not substantially greater than that of teenagers at baseline). By the end of the program, however, the medical students’ knowledge in this field increased as much as that of the pupils. In France, the core medical curriculum is 6 years long, and reproductive health is not taught until the 5^th^ year. This might therefore explain the absence of a drastic difference between pupils and medical students at baseline. Overall female’s knowledge (medical student or pupil’s) was higher than male’s. Difference in gender sexual health behaviors have been previously reported [9], but difference between knowledge poorly studied. Male are often excluded from sexual health program especially those concerning contraception’s. This difference in our study can also be linked with our questionnaire focused on contraception. Nevertheless young male should know as much as female’s to avoid unintended pregnancy.

Medical students were satisfied with their participation in peer-led education. As previously reported, their self-confidence increased [6]. A pedagogy comprising a theoretical course followed by a practical approach is recognized as valuable. Their opportunity to engage in a practical educational public health and preventive care project during the period of core studies was considered refreshing, as it differed from the traditional care [10,11]. It is possible that early exposure to preventive medicine may further encourage careers in this field. However, the design of our study did not assess the long-term impact of such program.

Whether the pupils’ and medical student’s increased knowledge has an impact on their behaviours has not yet been fully examined. Most studies have reported conflicting results, with end points frequently restricted solely to increased knowledge [3].

The long-term effects of such programs on sexual behaviours, rate of unwanted pregnancies or incidence of sexually transmitted infections is difficult to evaluate. The RIPPLE study assessed the impact over a seven-year period and found little difference when compared with conventional programs [5,12-14]. The reasons for mixed results are multiple: short intervention times, heterogeneity of peer educators, short follow up and the lack of subsequent review of the information given. Comparison among programs is methodologically hazardous. The content of different programs and interventions are generally poorly reported and studied populations are dissimilar for extrapolating results [13,15,16]. Our experimental program has the originality to gather various health care professionals implicated in reproductive health care. Particularly the family planning, a cost-saving preventive health structure, participates in the formation of medical students [17]. It may be representative of the various points of views involved in preventive and educational actions, though it was not compared to traditional teacher led interventions set by the ministry of education.

The experiences of other countries may influence future educational programs in reproductive health. The Netherlands has, for example, one of the lowest abortion rates in Europe [18]. This country promotes sex education as early as at the primary school level. Peer-to-peer education seems to be cost effective and has a degree of effectiveness after only a few sessions. Beside the time, contents and the early delivery of the information on sexual health problems it seems that repeat interventions have a positive impact [18].

Nonetheless, the present study has several limitations. Our data was collected from 65% of pupils who took the course. Therefore we have no information concerning the no responders whose knowledge may not have increased as a result of the program. Moreover there is a possible tutor effect according our analysis in cluster. Our study gives no information on the course’s impact on the sexual behaviour of the pupils and medical students. Initially, the aim was to evaluate the pupils’ sexual behaviour (i.e. unsafe sexual intercourse), but the national health education authority did not allow us to distribute a questionnaire asking about personal sexual habits. Therefore, we decided to evaluate only the knowledge level of the participants.

One of the advantages of peer education is that youth peer educators are less likely to be seen as authority figures [19]. In our program, medical students were older than pupils (by an average of seven years), this can limits the impact of our program on pupils. Inversely the young age exposed to low knowledge, errors or misunderstanding during question answers.

Our program, like other previously described models, is an example of how medical institutions can develop a collaborative community education project contributing to the education of medical students and secondary school pupils. Furthermore, the International Federation of Medical Students Associations has been working with WHO to improve medical students’ training on HIV/AIDS [20].

Negative effects of such health programs have not been described. There has been mention of resultant high-risk behaviour, but this has not been scientifically proven. Education programs cannot solve the whole problem. There are other factors that also have great influence on sexual behaviour, such as personal individual characteristics, family education and religious beliefs.

## Conclusions

Our study shows that the program was effective in increasing the knowledge of medical students as well as secondary school pupils. Male sexual health knowledge should be support.

## Abbreviations

IFR: Institut Federatif de Recherche; AIDS: Acquired immune deficiency syndrome; HIV: Human immunodeficiency virus.

## Competing interests

We have no financial relationship with the organization that sponsored the research. We have had the full control of all primary data and that we agree to allow the Journal to review our data if requested.

## Authors’ contributions

FB designed the project and the study, she wrote the manuscript. RS and LB conceived the study and helped to draft the manuscript. JB participate into data analysis and manuscript arrangements. ST checked for quality data and performed the statistical analysis. MCM coordinate the study. JD and CR participate into medical student formations. JD coordinates the link between medical school and national health education. George Leonetti coordinates medical student formation and authorized them to teach in schools. All authors read and approved the final manuscript.

## Pre-publication history

The pre-publication history for this paper can be accessed here:

http://www.biomedcentral.com/1472-6920/14/162/prepub
